# Barriers to Early Initiation of Breastfeeding: A Hospital-Based Qualitative Study

**DOI:** 10.24248/eahrj.v9i1.825

**Published:** 2025-09-30

**Authors:** Davis Rubagumya, Muzdalfat Abeid, Eric Aghan, Mariam Noorani

**Affiliations:** a Department of Family Medicine-Aga Khan University, Tanzania; b Department of Paediatrics – The Aga Khan University, Tanzania; c Department of Obstetrics and Gynaecology – The Aga Khan University, Tanzania; d Department of Family Medicine-Premier Care Clinic.

## Abstract

**Background:**

Breastfeeding is a key intervention to improve global targets on nutrition, health and survival. The World Health Organization recommends initiating breastfeeding within the first hour of birth to prevent infections, strengthen neonatal-maternal bonding, maintain thermoregulation and promote long term breastfeeding success. Global prevalence of early initiation of breastfeeding is 46% while in Tanzania it is 70%. The target set by the Baby Friendly Hospital Initiative guidelines for hospital deliveries is at least 80%.

**Objectives:**

This study aimed to explore barriers to early initiation of breastfeeding in a hospital setting using a descriptive qualitative approach.

**Methods:**

Semi-structured individual interviews were conducted to explore barriers to early initiation of breastfeeding from the perspective of midwives and post-partum mothers. Data was analysed using Systematic Text Condensation as described by Malterud.

**Results:**

Participants perceived that inadequate and sometimes conflicting information during antenatal period, especially on ideal time to start breastfeeding contributed to delayed initiation. Post-delivery practices such as perineal tear repair, along with environmental factors, including perceptions of unclean labour rooms and the presence of male personnel in the delivery room further hindered timely initiation. Overall, barriers were linked to gaps in knowledge, non-conducive postpartum environments and restrictive hospital practices.

**Conclusions:**

Improving early initiation of breastfeeding requires policies and programs aimed at strengthening the provision of breastfeeding related education to mothers during antenatal visits and enhancing the knowledge of midwives. At the health facility level, creating a supportive post-partum environment with individualised care, and implementing evidence-based practices are essential for promoting timely initiation.

## BACKGROUND

Breastfeeding provides optimal, safe and sustainable nutrition for newborns. Globally, an estimated 820,000 deaths of children under the age of five could be avoided every year through optimal breastfeeding practices.^1^ The United Nations Children's Fund (UNICEF) and World Health Organization (WHO) recommend initiating breastfeeding within the first hour post-delivery and continuing exclusive breastfeeding for the first 6 months.^2^

Colostrum, the first milk produced after delivery, is the perfect nutrition for newborns. It contains nutrients such as epidermal growth factor that help in intestinal maturation and repair, clearing excess bilirubin, modelling the immune system, prevention of infections and in neurodevelopment.^3^ Early initiation of breastfeeding exposes the newborn to maternal skin flora which help in colonising the infant's gut and skin while protecting against harmful bacteria.^4^ This practice also stabilises newborn's heart rate, temperature and breathing. Early initiation of breastfeeding has also been shown to predict subsequent breastfeeding success and exclusive breastfeeding ^5^.

Globally, 46% of newborns start breastfeeding within the first hour of birth^6^ while in Tanzania, the rate is higher at 70%, according to the 2022 Tanzania Demographic and Health Survey and malaria indicator survey.^7^ Several factors influencing early initiation of breastfeeding have been identified including delivery in health care facilities. A report by WHO and UNICEF involving 58 countries showed an increase in institutional deliveries from 53% in 2005 to 71% in 2017; parallel with a rise in early initiation of breastfeeding from 45% to 51%.^8^ However, the lower rate of increase in early initiation of breastfeeding indicates missed opportunities. A systematic review that examined facilitators and barriers to early and exclusive breastfeeding in health facilities in Sub-Saharan Africa identified facility infrastructure, supplies and staffing as barriers.^9^

The mother's level of education may also influence breastfeeding practices. Results from existing literature show conflicting results with some reporting positive association between higher education and early breastfeeding initiation, while others show the opposite. For instance, an Iranian study^10^ found that women with lower level of education, such as diploma level, were more likely to initiate breastfeeding early as compared to those with a university education^10^ Conversely, a study from Nepal revealed that women who had not attended school delayed breastfeeding initiation as compared to those who had formal education.^11^ Similarly, a secondary analysis of demographic and health surveys (DHS) of 16 Sub-Saharan African countries showed that mothers who completed primary education were more likely to initiate breastfeeding early as compared to those without formal education. Interestingly, mothers with secondary school education were not significantly different from those without formal education in terms of early initiation of breastfeeding.^12^ In contrast, another analysis of DHS data from 11 African countries showed that any level of education; whether primary, secondary or higher resulted in higher odds of early breastfeeding initiation compared to no formal education.^13^ These conflicting findings about the role of formal education in breastfeeding success warrant further exploration.

Antenatal clinic visits are recognised as an important avenue to impart breastfeeding education, however, an analysis of Demographic and Health Survey data from Gambia showed no association between antenatal clinic attendance and early initiation of breastfeeding.^14^ This suggests a possible missed opportunity for breastfeeding counselling during antenatal visits. In contrast, a meta-analysis from Ethiopia found that receiving health education during antenatal care promoted early initiation of breastfeeding. The same study also identified postpartum breastfeeding support as an important determinant of breastfeeding initiation.^15^

Mode of delivery also influences early initiation of breastfeeding, with vaginal births more likely to be followed by early initiation compared to caesarean delivery^16^. A meta-analysis from Ethiopia showed that women who delivered by caesarean had 79% lower odds of early initiation as compared to those delivering vaginally.^17^ Reasons mentioned for the delay in case of caesarean delivery include physical separation of infant and mother during recovery time, anaesthesia effect, restricted mobility, and distressful condition of neonate and critical condition of the mother after caesarean delivery. Nonetheless, evidence from quality improvement studies in India and Tanzania demonstrate that successful skin to skin contact and early initiation of breastfeeding can still be achieved in the operating theatre after caesarean delivery.^18, 19^

Cultural beliefs are known to impact early initiation of breastfeeding. In some cultures, colostrum is considered as medicine while others consider it useless milk or even harmful for the newborn. A qualitative study in Kenya, for instance, revealed that colostrum was considered dirty and therefore expressed and discarded during the first 2 days post-delivery.^20^ Conversely, a qualitative study from the Democratic Republic of Congo reported that most mothers fed their babies colostrum because they believed it contains vitamins and protects the baby from infections.^21^ In Tanzania, most studies have focused on exclusive breastfeeding, with relatively few addressing early initiation. One study from rural Tanzania found that facility-based delivery and vaginal birth were associated with early initiation^22^, while another study from Moshi showed an association between maternal knowledge of timely initiation and its actual practice.^23^ Notably, these Tanzanian studies, like much of the current global literature on breastfeeding initiation, are quantitative in nature. A qualitative approaching, however, is crucial to understand individual and systemic barriers within the health care facility context.

Although the prevalence of early initiation of breastfeeding in Tanzania is higher than the global average, it remains below the 80% mark recommended by the baby-friendly hospital initiative. To improve rates of early initiation of breastfeeding in Tanzania, it is important to identify barriers that contribute to suboptimal uptake.

The present study aimed to explore these barriers by examining the perspectives and experiences of mothers and midwives in a hospital maternity ward setting.

## METHODS

### Study Design

We conducted a qualitative study using individual interviews with key informants to explore barriers to early initiation of breastfeeding among healthy term neonates in an urban hospital setting. Qualitative description was selected as an appropriate method to answer the research question at hand focusing on experiences of midwives and nursing mothers who are directly involved in early initiation of breastfeeding.^24^

### Study Setting

The study was conducted at the Aga Khan Hospital, the largest private tertiary hospital in Tanzania. This not-for-profit, Joint Commission International (JCI) accredited hospital serves a mid to high socio-economic population. Although not formally certified as a Baby Friendly Hospital, staff follow the Baby Friendly Hospital Initiative recommended practices, including maintaining a written breastfeeding policy, encouraging rooming in, skin to skin contact and providing antenatal breastfeeding education, primarily through playing videos on a large screen. During the study period, there was no clear policy on early skin to skin contact and early initiation of breastfeeding. Immediate breastfeeding support was dependent on the midwife attending the delivery. Postnatally, nurses assess and support breastfeeding and formula milk is only given upon prescription by a paediatrician. One paediatrician, an International Board-Certified Lactation Consultant (IBCLC), provides specialised lactation support to mothers and infants facing breastfeeding challenges.

### Sample

The study involved midwives and mothers who had delivered healthy term neonates vaginally. Mothers who had postpartum complications were excluded. Purposive sampling method was used to recruit participants^25^ whereby potential participants were approached by the primary investigator and informed about the study; those who were willing to discuss their perspectives and share their experiences were recruited. No compensation was provided for participation. The estimated sample size was 12 participants, based on the concept of information power, which considers factors such as the study aim, specificity and analysis strategy^26^. For this study, the aim was narrowed to early initiation and the participants were specifically nurses and mothers who are involved in the process of early initiation, hence a smaller sample size was anticipated. Final sample size was determined by data saturation. No new themes emerged after interviewing three nurses and five mothers. One additional mother was interviewed to confirm saturation, after which the sample was deemed sufficient to address the research question.

### Data Collection

Data was collected between 2^nd^ November and 3^rd^ December 2019. Written study information in both English and Swahili was provided to all potential participants, allowing adequate time for questions, followed by obtaining informed written consent prior to the interview.

The primary researcher (DR) conducted the interviews. At the time, he was the senior resident in family medicine. He is a Tanzanian with no fixed beliefs about breastfeeding. He had no supervisory relationship with any of the participating staff and was not involved directly in the care of the mothers who were interviewed. He was coached on the interview process by the supervisor (MA) who is a seasoned qualitative researcher. The coaching involved a theoretical discussion on how to approach a qualitative interview followed by a practical demonstration and simulation activity.

All interviews with mothers were conducted within 48 hours of delivery. To ensure comfort, mothers were approached when the neonate was asleep and a family member was present. Midwives were interviewed during their off days to avoid compromising patient care. Interviews were conducted in Swahili in a private room within the post-natal unit using a semi-structured interview guide. Each interview lasted approximately 30 minutes and was audio recorded. The audio files were transcribed and translated into English by the researcher. The English transcripts were then reviewed by an International English Language Testing System (IELTS) certified Swahili speaker for grammatical accuracy and back-translated into Swahili to verify consistency with the original version. No significant mismatches were encountered.

The audio files recorded were stored in a password protected Universal Serial Bus flash drive and the primary researcher was responsible to transfer recorded information from the device to the password protected computer and hard drive.

### Data Analysis

No software was employed during the analysis process as data was handled manually. Data was analysed by the primary researcher (DR) with frequent consultation with the co-investigators (MN, MA and EA). Systematic Text Condensation, as described by Malterud, was applied to analyse the collected data.^27^ This approach allowed for a thorough examination of the manifest content, revealing challenges to early initiation of breastfeeding from the perspective of mothers and midwives. The analysis involved multiple readings of the English transcripts to capture the emerging themes and meaning units, providing an overall impression of the data. The sorted meaning units were coded and grouped into categories and subcategories which were stamped at the manifest level and ratified against the original transcripts to ensure accuracy and consistency.

### Trustworthiness of the Study

To maintain credibility, multiple data reviews were done. The primary researcher and supervisors independently interpreted the data, resulting in the development of overall categories. Areas of uncertainty in the transcripts were cross-checked with respective participants to enhance common agreement. Cross checking was done through a face-to-face discussion with a specific midwife during her shift for clarification of transcribed points for common understanding. For the mothers, cross checking was not feasible since they had been discharged.

Transferability was enhanced by providing detailed description of the study setting, design, participants, data collection methods and findings, allowing readers to contextualise the results. Emerging categories were presented in a way that enables flexibility in reaching conclusions.

Dependability was maintained by clearly documenting all steps of the study and noting encountered limitations. Variability in the findings was expected since the study focused on a range of experiences related to early initiation of breastfeeding.

### Ethical Considerations

Ethical approval for the study was obtained from the Aga Khan University Ethics Review Committee (AKU-ERC), Ref no.: AKU/201e/30s/Jb. Permission to conduct the study was also granted by the Hospital's Medical Director's office. Informed written consent was obtained from all participants after they had received adequate information about the study and sufficient time to consider participation. Confidentiality was maintained throughout the process of data collection, storage, and management.

## RESULTS

A total of 6 mothers were interviewed, aged between 18 and 40 years. Two were first time mothers while 4 had delivered their second child. They were all married and had attained at least a diploma level education. Three nurses who participated in the study and were aged 31 years and above. Two had a diploma in nursing while 1 had a bachelor's degree. Their experience in the profession ranged from 5 to 20 years. The demographic characteristics are summarized in Table 1

**TABLE 1:** Baseline Characteristics of Study Participants

Variable	Nurses	Mothers
Age (Years)		
18–30		3
31–40	1	3
>40	2	
Parity		
1	1	2
2	1	4
5	1	
Marital Status		
Married	3	6
Occupation		
Employed worker	3	2
Trader		4
Education		
Diploma	2	2
Degree	1	4

Data analysis yielded three main categories, which are highlighted in Table 2. The relevant explanations and quotes are described in the texts below.

**TABLE 2:** Categories and Subcategories Derived from the Analysis

Categories	Subcategories
Knowledge gaps & conflicting information	Conflicting information from different sources Information on breastmilk substitutes Inadequate antenatal education Unawareness on the recommended time for initiation
Immediate post-delivery events as barriers to breastfeeding	Genital tears repair Routine post-delivery practices
Non conducive breastfeeding environment	Lack of care provider support Mothers’ readiness to breastfeed Male presence in the delivery room Dirty surroundings

### Knowledge Gaps and Conflicting Information

All participants perceived insufficient and conflicting information as a hindrance to early initiation of breastfeeding. Moreover, they described that inadequate information about breastfeeding during antenatal clinic visits often contributed to delay in initiation of breastfeeding. Within the category, 4 sub-categories emerged

**Conflicting information from different sources:** Participants felt that information received on breastfeeding from various sources was confusing with both breastmilk and formula milk being advocated for newborn babies. One mother said that *“I feel it's good to be informed because sometimes you might come with street information and your mind is just thinking about formula”* (Mother 2)

**Information on breastmilk substitutes:** Participants highlighted the overwhelming availability of information on formula milk compared to breastfeeding, particularly through social media, as a major barrier to early initiation of breastfeeding after giving birth. This abundancy of information created expectations among some women that formula milk would be readily available after birth, reducing their motivation to initiate breastfeeding. As one mother explained, *“I hear about formula milk more than breastmilk”* (Mother 6) Another mother said, *“I can tell you; a mother will come knowing there is alternative to breastmilk, hence won't bother with breastfeeding.”* (Mother 4)

**Inadequate antenatal education:** Participants consistently described insufficient breastfeeding education during antenatal visits as a barrier to early initiation of breastfeeding. Participants felt that clinic visits should incorporate detailed breastfeeding lessons on top of assessing the unborn baby's wellbeing. A nursing mother expressed this by saying *“hmm! The doctor mostly would enquire about the age of pregnancy, laboratory results, listening to the baby's heartbeats, but concerning breastfeeding, no information about that.”* (Mother 2)

Nurses also emphasised the lack of individualised breastfeeding guidance, particularly for mothers with special needs, as one noted, *“You might find a woman has seven visits and has flat nipple, meaning no one informed her even about preparing for breastfeeding despite such a condition”.* (Nurse 2) Another nurse expressed frustration at the lack of preparation: *“Sometimes it's frustrating, you find a lady having (‘sifuri’) zero knowledge on breastfeeding and has a documentation of six clinic visits”* (Nurse 3)

**Unawareness of the Recommended Time for Initiation:** Participants reported varying and sometimes inaccurate understanding of appropriate timing for initiating breastfeeding, reflecting lack of consistent knowledge among mothers and care givers. One mother believed that initiation could take place within several hours after delivery: *“Time should be within three hours, the mother has to be back in the ward and comfortable”* (Mother 2). In contrast, a nurse admitted uncertainty about a defined timeframe, stating, *“Hmm! I have never heard of such a time. (with confidence), however it is supposed to be immediately, but about time, even in books, that is not there”* (Nurse 2) Another mother stressed the importance of clarity, noting that awareness of the one-hour recommendation would empower mothers to advocate for timely initiation: “*I need to know the time, I never knew that, khaa!! if you know that immediately means within one hour, reminding a nurse after delivery becomes easy*”. (Mother 6)

### Immediate Postdelivery Events as Barriers to Breastfeeding

Participants expressed concerns about routine postdelivery practices and their impact on early initiation of breastfeeding. Commonly mentioned practices included the admission of newborns weighing over 4 kilograms to the neonatal unit for observation, and the immediate placement of newborns in baby warmers. Such interventions were perceived as delaying the opportunity for mothers to initiate breastfeeding promptly. Two sub-categories emerged under this theme:

**Genital tears repair:** Participants reported that perineal tear repair process hindered early initiation of breastfeeding since a lot of time is consumed in preparing instruments as well as performing the procedure. One nurse reported, *“Even for those who deliver normally, babies might miss the chance to breastfeed immediately, for example, in case a lady had a tear, this can hamper breastfeeding”* (Nurse 1). However, not all participants viewed this as an insurmountable obstacle. One mother suggested that breastfeeding could still be initiated despite the perineal tear with the assistance of the attending nurse *“I believe that nurse would even help me to breastfeed even before starting the tear repair process”*. (Mother 3)

**Routine post-delivery practices:** Some routine newborn care practices were described as barriers to early initiation of breastfeeding. For example, nurses explained that newborns weighing more than 4 kilograms are often observed in the neonatal unit to prevent hypoglycaemia, a process that may involve introducing formula feeding. One nurse explained: “*We provide information that the place for observation will be in the nursery (for big babies) and the baby will be prescribed formula milk (as initial food)*” (Nurse 2). Similarly, mothers reported that immediate placement of babies in warmers after delivery, followed by other nursing procedures, often disrupted the opportunity to breastfeed, as one mother recounted: “*Hmmm! no! my baby was put on a warmer, and that was it in there*”. (Mother 1)

## NON-CONDUCIVE BREAST-FEEDING ENVIRONMENT

Participants expressed concerns that the post-delivery environment was not conducive for initiating breastfeeding. 4 sub-categories emerged: lack of care provider support, the mother's readiness, the presence of males in the delivery room, and the physical surroundings.

**Lack of care provider support:** Mothers pointed out the absence of midwife support as a barrier to early initiation of breastfeeding. Midwives on the other hand cited heavy workloads and competing responsibilities as reasons for their limited assistance, as one nurse noted, *“Sometimes documentation can consume your time, and you may even forget to assist a lady to breastfeed, but this depends on how busy the day has been.”* (Nurse 1) Similarly, another nurse lamented, *“Human resource also matters because some days where there are many deliveries, you might feel you are going nuts, hence it's tough to help mothers start breastfeeding well!!”* (Nurse 2)

**Mother's readiness to breastfeed:** Fatigue and discomfort after labour influenced mothers’ ability and willingness to initiate breastfeeding. One mother said: “*I was tired lol, to start breastfeeding within the labour room is a big NO*”. (Mother1). Another sentiment from a mother, *“(Alaa!), labour pain hmm!!!!!! you find that a woman is tired after delivery, so it becomes tough to start breastfeeding”* (Mother 5). The perception that breastfeeding should occur when a mother is relaxed and in good mood also emerged. One nurse shared her perspective; *“Sometimes mothers are tired after delivery because of labour process, hence are not relaxed to start breastfeeding.”* (Nurse 3).

**Male presence in the delivery room:** Cultural and social dynamics were also reported to affect breastfeeding. One nurse said: *“It's not possible to breastfeed in presence of a man, even if it's the husband who is around.”* (Nurse 3). Cultural influence was mentioned by another nurse, *“Sometimes culture, for instance, among Chaggas (a community from Kilimanjaro region in Northern Tanzania) it's difficult to breastfeed in front of people you are not close with. Hence depending on who are the people within labour room, this can be a challenge.”* (Nurse 1)

**Dirty surroundings:** Participants reckoned that the labour room environment after delivery discourages initiation of breastfeeding, with one mother saying, *“You know it's hard to start breastfeeding in labour room without being comfortable, and after being cleaned, comfortability actually comes, and that is when breastfeeding is possible’* (Mother 2). One nurse spoke of blood: *“The environment with blood makes mothers hesitate to breastfeed, some of them say that the environment kind of reminds them of labour pain, hence for them to relax and breastfeed, they want to be in the ward!”* (Nurse 2)

## DISCUSSION

The present study provides insights on the barriers to early initiation of breastfeeding, drawing on the experiences and perspectives of midwives and immediate postpartum mothers.

Inadequate information provided during antenatal clinic visits emerged as a major limitation to early initiation of breastfeeding. Similar findings have been reported in different studies including one from Uganda^28^, which showed that inadequate prenatal guidance was associated with 3 times higher odds of delaying initiation of breastfeeding. However, it is worth noting that regular attendance of antenatal clinics does not guarantee successful initiation of breastfeeding as demonstrated by a study from Gambia.^14^ It is therefore the quality of activities and education provided during these visits that matters most.

The Tanzanian antenatal care guideline specifies that breastfeeding counselling should be done during the last antenatal visit as adapted from the WHO guideline on focused antenatal care.^29^ However, our findings highlight that specific guidance on the timing of breastfeeding initiation is often missing. This gap underscores the need for healthcare providers to broaden the scope of information offered to pregnant women, with particular attention to early initiation. In addition, incorporating a woman's previous breastfeeding history and performing breast examinations to identify women who are likely to have challenges in breastfeeding is crucial. Further studies involving both pregnant women and antenatal care providers is warranted to better understand the challenges of providing effective breastfeeding education during the antenatal period.

Easy availability of information on breast milk substitutes also emerged as a significant barrier in the present study.

This was also reported in a study from South Sudan which showed that mothers were two times more likely to fail in the practice of early initiation of breastfeeding after exposure to breast milk substitutes adverts.^30^ Such advertising contravenes the World Health Organisation's code of marketing of breast milk substitutes, whose violation has been documented globally, particularly through social media marketing^31^, with over 60% of pregnant women and mothers of young children reporting exposure to promotions of products covered under the code.

A surprising finding from this study was lack of awareness of the ideal time to initiate breastfeeding among health care providers. This raises concern about the education and training of nurses and midwives, an area that requires further exploration. Without adequate knowledge, health workers cannot implement guidelines or provide the required support to postpartum mothers. A review of the nursing curricula may be warranted to access the extent of lactation-related content, while continuing education opportunities could incorporate targeted knowledge and skills to bridge this gap. Given that health worker support; both prenatally and immediately post-partum, is strongly associated with successful breastfeeding, addressing this deficiency is crucial to improving early initiation outcomes.^32^

Maternal knowledge was also limited in our study. Awareness of the appropriate time to initiate breastfeeding can positively influence outcomes by enabling mothers to work collaboratively with care providers during the immediate postpartum period. Supporting this, a cross-sectional, health facility-based survey in Saudi Arabia revealed that knowing the right time to start breastfeeding among mothers was a positive influencer of early initiation of breastfeeding.^33^

Mode of delivery influences early initiation ofbreastfeeding with spontaneous vertex delivery within hospital showing positive impact as compared to caesarean section. However, our findings revealed that even after vaginal delivery, certain hospital practices delayed breastfeeding initiation. These included postpartum procedures such as; perineal tear repair, placing babies on warmers after delivery or protocols mandating that macrosomic infants (> 4 kg) be admitted to the neonatal unit for observation and formula feeding. Although macrosomic infants may be at risk of hypoglycaemia, current recommendations emphasize immediate skin to skin contact and early initiation of breastfeeding.^34^ Individualised postnatal care, tailored to both maternal and neonatal conditions, is therefore essential.

The conduciveness of the postpartum environment also emerged as a barrier in our study. Participants cited inadequate staff support, environmental cleanliness, and the presence of other people in the delivery space as factors influencing initiation of breastfeeding. Lack of support from staff could be due to shortage of staff or a heavy workload which limits the ability of the midwives to provide timely support to mother. This observation aligns with reports of a Tanzanian study which highlighted staff shortage as a key challenge for midwives in urban settings.^35^ A systematic review which explored barriers and facilitators to early and exclusive breastfeeding in health facilities in Sub-Saharan Africa also highlighted health facility infrastructure and staffing as growing concerns in breastfeeding support.^9^

Unique cultural and contextual factors also shaped breastfeeding practices. Mothers reported barriers such as unclean, dirty labour room and presence of male personnel, issues rarely discussed in prior literature. Recognition and acceptance of the cultural context in which breastfeeding occurs is essential for designing appropriate interventions. Cultural practices differ globally and are unique to different countries and settings. In Turkey, for example, a qualitative study of midwives reported cultural practices like feeding newborn babies dates and a patriarchal family structure as barriers to early initiation of breastfeeding.^36^

Finally, maternal fatigue was frequently reported as a limiting factor. This can, however, be mitigated through the support of a close relative or birth companion during labour, who can facilitate breastfeeding by providing support to the mother. Support from an intrapartum birth companion of choice was found to reduce time to early initiation of breastfeeding in a quality improvement project conducted in India.^37^

## CONCLUSIONS

This study highlights barriers to early initiation of breastfeeding based on the experiences and perspectives of midwives and postpartum mothers in a private urban hospital in a low- to middle income country. The main barriers identified were; gaps in knowledge, immediate postpartum practices and individual perceptions of a non-conducive environment.

To overcome these barriers, more focus is needed on antenatal education, staff training and individualised post-partum care, while taking cultural practices into account. At policy level, a standardised program for antenatal breastfeeding education needs to be developed and implemented across all antenatal clinics, it should include early initiation of breastfeeding. The nursing and midwifery curricula should be reviewed and strengthened to include comprehensive breastfeeding knowledge and skills.

At the hospital level, adoption of evidence-based practices in the management of macrosomic infants is necessary to prevent unnecessary delays and promote timely breastfeeding initiation. Further studies involving both pregnant women and ANC providers can provide deeper insight into the challenges of delivering effective antenatal breastfeeding education. Additionally, studies using implementation science approach can be implemented to find the best ways to integrate early initiation into routine practice.

### Study Limitations

The study has some limitations. All participating mothers had at least a diploma-level education, which may limit the applicability of findings to women with lower educational background. Furthermore, as the study was conducted in an urban private hospital, the findings may not be fully transferable to public or rural hospital settings. Finally, the use of purposive sampling may have introduced selection bias and may also impact transferability to the wider population.

## References

[1] Victora, C. G., Bahl, R., Barros, A. J., França, G. V., Horton, S., Krasevec, J., Murch, S., Sankar, M. J., Walker, N.,& Rollins, N. C. (2016). Breastfeeding in the 21st century: epidemiology, mechanisms, and lifelong effect. The lancet, 387(10017), 475–490. 10.1016/S0140-6736(15)01024-7 26869575

[2] World Health Organisation. (2018). Implementation guidance: protecting, promoting and supporting breastfeeding in facilities providing maternity and newborn services – the revised Baby-friendly Hospital Initiative. 29565522

[3] Ballard, O., & Morrow, A. L. (2013). Human milk composition: nutrients and bioactive factors. Pediatric Clinics, 60(1), 49–74. 23178060 10.1016/j.pcl.2012.10.002 PMC3586783

[4] UNICEF. (2016). FROM THE FIRST HOUR OF LIFE Making the case for improved infant and young child feeding everywhere.

[5] Permatasari, T. A. E., & Syafruddin, A. (2016). Early initiation of breastfeeding related to exclusive breastfeeding and breastfeeding duration in rural and urban areas in Subang, West Java, Indonesia. Journal of Health Research, 30(5), 337–345.

[6] UNICEF. (2025). Global Breastfeeding Scorecard 2024. Retrieved from https://knowledge.unicef.org/child-nutrition-and-development/resource/global-breastfeeding-scorecard-2024

[7] Ministry of Health (MoH) [Tanzania Mainland], Ministry of Health (MoH) [Zanzibar], National Bureau of Statistics (NBS), Office of the Chief Government Statistician (OCGS), and ICF. (2022). Tanzania Demographic and Health Survey and Malaria Indicator Survey.

[8] UNICEF, WHO. (2018). Capture the Moment – Early initiation of breastfeeding: The best start for every newborn.

[9] Kinshella, M. L. W., Prasad, S., Hiwa, T., Vidler, M., Nyondo-Mipando, A. L., Dube, Q., Goldfarb, D., & Kawaza, K. (2021). Barriers and facilitators for early and exclusive breastfeeding in health facilities in Sub-Saharan Africa: a systematic review. Global Health Research and Policy, 6, 1–11. 10.1186/s41256-021-00206-2 PMC8259208 34229756

[10] Haghighi, M., & Taheri, E. (2015). Factors associated with breastfeeding in the first hour after birth, in baby friendly hospitals, Shiraz-Iran.

[11] Acharya, P., & Khanal, V. (2015). The effect of mother's educational status on early initiation of breastfeeding: further analysis of three consecutive Nepal Demographic and Health Surveys. BMC Public Health, 15, 1–12. 26482789 10.1186/s12889-015-2405-y PMC4610048

[12] Wako, W. G., Wayessa, Z., & Fikrie, A. (2022). Effects of maternal education on early initiation and exclusive breastfeeding practices in sub-Saharan Africa: a secondary analysis of Demographic and Health Surveys from 2015 to 2019. BMJ open, 12(3), e054302. 10.1136/bmjopen-2021-054302 PMC8928244 35292494

[13] Ameyaw EK, Adde KS, Paintsil JA, Dickson KS, Oladimeji O, Yaya S. Health facility delivery and early initiation of breastfeeding: Cross-sectional survey of 11 sub-Saharan African countries. Health Science Reports. 2023 May;6(5):e1263. 37181665 10.1002/hsr2.1263 PMC10173260

[14] Darboe, M. L., Jeyakumar, A., Mansour, S. M., & Valawalkar, S. (2023). Determinants of early initiation of breastfeeding in The Gambia: a population-based study using the 2019–2020 demographic and health survey data. International Breastfeeding Journal, 18(1), 33. 37349805 10.1186/s13006-023-00570-4 PMC10288753

[15] Kebede SD, Forster EM, Agmas K, Aytenew TM, Creedy DK. Prevalence and contributing factors of early initiation of breastfeeding (EIBF) in Ethiopia: a systematic review and meta-analysis. BMC Public Health. 2025 Apr 12;25(1):1377. 40221719 10.1186/s12889-025-22568-9 PMC11992844

[16] Karim, F., Khan, A. N. S., Tasnim, F., Chowdhury, M. A. K., Billah, S. M., Karim, T., Arifeen, S. E., & Garnett, S. P. (2019). Prevalence and determinants of initiation of breastfeeding within one hour of birth: An analysis of the Bangladesh Demographic and Health Survey, 2014. PloS one, 14(7), e0220224. 31344069 10.1371/journal.pone.0220224 PMC6658080

[17] Getaneh, T., Negesse, A., Dessie, G., Desta, M., Temesgen, H., Getu, T., & Gelaye, K. (2021). Impact of cesarean section on timely initiation of breastfeeding in Ethiopia: a systematic review and meta-analysis. International breastfeeding journal, 16(1), 1–10. 34225731 10.1186/s13006-021-00399-9 PMC8259022

[18] Dudeja, S., Sikka, P., Jain, K., Suri, V., & Kumar, P. (2018). Improving first-hour breastfeeding initiation rate after cesarean deliveries: a quality improvement study. Indian pediatrics, 55, 761–764. 30345980

[19] Osman R, Katigula I, Charles R, Ayubu A, Mkakilwa P, Abdallah Y, Noorani M. Improving early initiation and exclusive breastfeeding practice through PDSA framework: the experience at a private hospital in Tanzania. BMJ Open Quality. 2024;13(4):e002893. 10.1136/bmjoq-2024-002893 PMC11881197 39675816

[20] Wanjohi, M., Griffiths, P., Wekesah, F., Muriuki, P., Muhia, N., Musoke, R. N., … & Kimani-Murage, E. W. (2016). Sociocultural factors influencing breastfeeding practices in two slums in Nairobi, Kenya. International breastfeeding journal, 12, 1–8. 28096888 10.1186/s13006-016-0092-7 PMC5225512

[21] Babakazo P, Piripiri LM, Mukiese JM, Lobota N, Mafuta É. Breastfeeding practices and social norms in Kinshasa, Democratic Republic of the Congo: A qualitative study. PLOS Global Public Health. 2024 Apr 16;4(4):e0000957. 38626214 10.1371/journal.pgph.0000957 PMC11020689

[22] Exavery, A., Kanté, A. M., Hingora, A., & Phillips, J. F. (2015). Determinants of early initiation of breastfeeding in rural Tanzania. International breastfeeding journal, 10, 1–9. 26413139 10.1186/s13006-015-0052-7 PMC4582933

[23] Lyellu, H. Y., Hussein, T. H., Wandel, M., Stray-Pedersen, B., Mgongo, M., & Msuya, S. E. (2020). Prevalence and factors associated with early initiation of breastfeeding among women in Moshi municipal, northern Tanzania. BMC pregnancy and childbirth, 20, 1–10. 10.1186/s12884-020-02966-0 PMC7216396 32393191

[24] Bradshaw, C., Atkinson, S., & Doody, O. (2017). Employing a qualitative description approach in health care research. Global qualitative nursing research, 4, 2333393617742282. 29204457 10.1177/2333393617742282 PMC5703087

[25] Palinkas, L. A., Horwitz, S. M., Green, C. A., Wisdom, J. P., Duan, N., & Hoagwood, K. (2015). Purposeful sampling for qualitative data collection and analysis in mixed method implementation research. Administration and policy in mental health and mental health services research, 42, 533–544. 24193818 10.1007/s10488-013-0528-y PMC4012002

[26] Malterud, K., Siersma, V. D., & Guassora, A. D. (2016). Sample size in qualitative interview studies: guided by information power. Qualitative health research, 26(13), 1753–1760. 26613970 10.1177/1049732315617444

[27] Malterud, K. (2012). Systematic text condensation: a strategy for qualitative analysis. Scandinavian journal of public health, 40(8), 795–805. 23221918 10.1177/1403494812465030

[28] Kalisa, R., Malande, O., Nankunda, J., & Tumwine, J. K. (2015). Magnitude and factors associated with delayed initiation of breastfeeding among mothers who deliver in Mulago hospital, Uganda. African health sciences, 15(4), 1130–1135. 26958013 10.4314/ahs.v15i4.11 PMC4765405

[29] World Health Organization. (2002). WHO antenatal care randomized trial: manual for the implementation of the new model (No. WHO/RHR/01.30).

[30] Bruno Tongun, J., Sebit, M. B., Mukunya, D., Ndeezi, G., Nankabirwa, V., Tylleskar, T., & Tumwine, J. K. (2018). Factors associated with delayed initiation of breastfeeding: a cross-sectional study in South Sudan. International breastfeeding journal, 13, 1–7. 30002722 10.1186/s13006-018-0170-0 PMC6034205

[31] Lutter, C. K., Hernández-Cordero, S., Grummer-Strawn, L., Lara-Mejía, V., & Lozada-Tequeanes, A. L. (2022). Violations of the International Code of Marketing of Breast-milk Substitutes: a multi-country analysis. BMC Public Health, 22(1), 1–11. 36514038 10.1186/s12889-022-14503-z PMC9749209

[32] Namasivayam, V., Dehury, B., Prakash, R., Becker, M., Avery, L., Sankaran, D., … & Isac, S. (2021). Association of prenatal counselling and immediate postnatal support with early initiation of breastfeeding in Uttar Pradesh, India. International breastfeeding journal, 16(1), 1–11. 33726797 10.1186/s13006-021-00372-6 PMC7968284

[33] Ahmed, A. E., & Salih, O. A. (2019). Determinants of the early initiation of breastfeeding in the Kingdom of Saudi Arabia. International breastfeeding journal, 14(1), 1–13. 30984282 10.1186/s13006-019-0207-z PMC6444675

[34] Academy of Breastfeeding Medicine. (2021). ABM clinical protocol# 1: guidelines for glucose monitoring and treatment of hypoglycemia in term and late preterm neonates, revised 2021. Breastfeeding Medicine, 16(5), 353–365. 33835840 10.1089/bfm.2021.29178.new

[35] Bremnes, H. S., Wiig, Å. K., Abeid, M., & Darj, E. (2018). Challenges in day-to-day midwifery practice; a qualitative study from a regional referral hospital in Dar es Salaam, Tanzania. Global health action, 11(1), 1453333. 29621933 10.1080/16549716.2018.1453333 PMC5912436

[36] Ceylan, S. S., & Çetinkaya, B. (2020). Views of Maternity Nurses Relating to Barriers in Early Initiation of Breastfeeding: A Qualitative Study. Journal of Pediatric Research.

[37] Gadappa, S. N., & Deshpande, S. S. (2021). A Quasi-Experimental Study to Compare the Effect of Respectful Maternity Care Using Intrapartum Birth Companion of Her Choice on Maternal and Newborn Outcome in Tertiary Care Centre. The Journal of Obstetrics and Gynecology of India, 71, 84–89. 34924719 10.1007/s13224-021-01587-7 PMC8633154

